# Supplementary material for: A Critical View of the Application of the APEX Software (Aqueous Photochemistry of Environmentally-Occurring Xenobiotics) to Predict Photoreaction Kinetics in Surface Freshwaters
Source: Molecules. 2019 Dec 18;25(1):9. doi: 10.3390/molecules25010009 (PMC7017383; doi:10.3390/molecules25010009)
Supplement: Supplementary file 1 [file molecules-25-00009-s001.zip › 3.2.4_gcc-4.4.0.zip!/3.2.4_gcc-4.4.0/3.2.4_gcc-4.4.0/doc/octave/html/interpreter/Private-Functions.html]

Private Functions - Untitled


Next: Overloading and Autoloading,
Previous: Subfunctions,
Up: Function Files


---

#### 11.7.3 Private Functions

In many cases one function needs to access one or more helper
functions. If the helper function is limited to the scope of a single
function, then subfunctions as discussed above might be used. However,
if a single helper function is used by more than one function, then
this is no longer possible. In this case the helper functions might
be placed in a subdirectory, called "private", of the directory in which
the functions needing access to this helper function are found.

As a simple example, consider a function `func1`, that calls a helper
function `func2` to do much of the work. For example

```
     function y = func1 (x)
       y = func2 (x);
     endfunction
```

Then if the path to `func1` is `<directory>/func1.m`, and if
`func2` is found in the directory `<directory>/private/func2.m`,
then `func2` is only available for use of the functions, like
`func1`, that are found in `<directory>`.
